# Machine Learning-Derived Risk Groups and Clinical Implementation of Survival Prediction in Lung Cancer: Evidence from a Kazakh National Cohort

**DOI:** 10.3390/diagnostics16101479

**Published:** 2026-05-13

**Authors:** Zeinep Avizova, Ayan O. Myssayev, Yerbolat M. Iztleuov

**Affiliations:** 1 Department of Radiology, West Kazakhstan Marat Ospanov Medical University, Aktobe 030019, Kazakhstan; iztleuov@zkmu.kz; 2 Department of Hygiene and Epidemiology, South Kazakhstan Medical Academy, Shymkent 160011, Kazakhstan; 3 Office of the Vice-Rector for Strategic Development and International Cooperation, Astana Medical University, Astana 010000, Kazakhstan; amyssayev@gmail.com

**Keywords:** lung neoplasms, machine learning, prognosis, random forests, risk stratification

## Abstract

**Background/Objectives**: Lung cancer remains a leading cause of cancer-related death, and prognostic assessment relies mainly on TNM staging, which incompletely captures patient heterogeneity. Machine learning (ML) methods may improve survival prediction, but their use in real-world national registries with rigorous validation remains limited. This study aimed to develop ML-derived phenotypes and 1-year mortality risk groups and to evaluate their performance and clinical utility in a national lung cancer cohort from Kazakhstan. **Methods**: We conducted a retrospective study using a national registry including 13,685 patients. Eight routinely collected predictors were analyzed. K-means clustering was used for exploratory phenotyping. A random survival forest (RSF) model estimated 1-year mortality risk and defined low, intermediate, and high risk groups. Performance was evaluated using temporal validation, cross-validation, and bootstrap correction. Discrimination was assessed using the concordance index, prediction accuracy using the Brier score, and calibration using risk group comparisons. Comparator models included penalized Cox and TNM-only models. Clinical utility was assessed using decision-curve analysis. **Results**: Two phenotypes showed distinct survival outcomes, although cluster separation was modest. The RSF model showed stable performance (C-index 0.679; corrected 0.663). Risk groups demonstrated strong survival separation (high vs. low: HR 5.66). The RSF model outperformed the penalized Cox (C-index 0.544) and TNM (0.606), with improved accuracy (Brier 0.169 vs. 0.212). Calibration was generally good. Decision-curve analysis showed greater net benefit. **Conclusions**: An RSF-based model using routine registry data provided robust internally validated risk stratification and improved predictive performance. Clustering results were exploratory. External validation is re-quired before clinical implementation.

## 1. Introduction

Lung cancer remains the leading cause of cancer-related mortality worldwide, despite advances in screening, diagnosis, and treatment [1,2]. In Kazakhstan, lung cancer represents a substantial public health burden and remains a major contributor to cancer-related mortality, with most patients still presenting at advanced stages of disease [3,4,5,6]. Prognosis is highly heterogeneous and depends on a complex interplay of tumor stage, histology, patient comorbidities, and health-system factors, yet risk assessment in routine practice still relies largely on TNM staging and a limited set of clinical variables [7,8]. Conventional survival models such as Cox proportional hazards regression have provided valuable insights but may struggle to capture nonlinear relationships and interactions in large, heterogeneous populations.

Machine learning (ML) methods have emerged as powerful tools for survival prediction in lung cancer, enabling the integration of multiple clinical, radiologic, and molecular features and often improving discrimination compared with traditional models [9,10,11]. Recent studies have applied algorithms such as random survival forests (RSFs), gradient boosting survival models, and deep learning-based approaches to predict overall survival or treatment outcomes in non-small cell and small cell lung cancer, frequently reporting higher concordance indices and more flexible handling of censored data. At the same time, unsupervised learning techniques have been used to identify latent clinical or radiomic phenotypes with distinct prognoses, suggesting that phenotype-based grouping may add value beyond conventional staging [12].

However, several important gaps remain in the current literature. Many published ML models are developed in single-center or trial-based cohorts and focus primarily on statistical performance rather than clinical implementation [7]. Decision-curve analysis (DCA) has increasingly been recommended as a complementary method to quantify the net clinical benefit of prediction models across a range of decision thresholds, yet it is still underused in lung cancer survival modeling [13]. In addition, rigorous internal validation approaches, such as temporal validation, cross-validation, or bootstrap resampling, are not always applied, raising concerns about overfitting and the reliability of reported performance [14]. Direct comparisons between machine learning models and traditional statistical approaches using identical predictors remain heterogeneous and inconsistently reported across studies, particularly in large real-world datasets, which complicates the interpretation of relative model performance [15,16,17,18]. Finally, there is a paucity of data from Central Asia, and it is unclear whether ML-based risk tools derived from Western or East Asian populations are directly applicable to the Kazakh context.

In this study, we used a national lung cancer registry from Kazakhstan to: (1) derive data-driven patient phenotypes using unsupervised ML, (2) develop a random survival forest model to estimate individualized 1-year mortality risk, (3) construct simple three-level ML risk groups for clinical use, and (4) evaluate their prognostic value and clinical utility compared with TNM stage using survival metrics and decision-curve analysis. Our aim was to propose a pragmatic, registry-based ML framework that can support risk-informed decision-making in lung cancer care in Kazakhstan and similar settings.

## 2. Materials and Methods

### 2.1. Study Design and Data Source

This retrospective cohort study used data from the national lung cancer registry of Kazakhstan. Ethical approval was obtained from the Bioethics Committee of West Kazakhstan Medical University (Protocol No. 3, 28 March 2025). Because anonymized registry data were analyzed, informed consent was waived.

**Figure 1:**
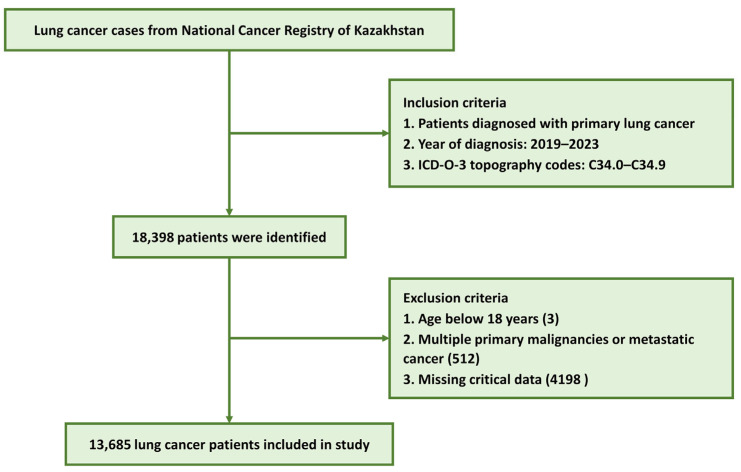
Flowchart of study cohort selection.

Patients newly diagnosed with lung cancer between 1 January 2019 and 31 December 2023 were eligible. The registry systematically collects standardized sociodemographic and clinical data for epidemiological surveillance and outcomes research. Registry records were linked with survival data to enable follow-up. A total of 18,398 patients were initially identified in the registry. After applying exclusion criteria, the final analytic cohort consisted of 13,685 patients. Specifically, 3 patients aged <18 years and 512 patients with secondary malignancies were excluded. Additionally, 4198 patients were excluded due to missing critical data required for survival analysis (Figure 1).

### 2.2. Outcome and Predictors

The primary outcome was overall survival (OS), defined as time from diagnosis to death from any cause. Survival time was measured in months, and patients alive at the end of follow-up or lost to follow-up were right-censored at the last known contact.

Eight routinely collected baseline predictors were analyzed: age, sex, nationality, residence (urban/rural), social status, diagnostic circumstances, histology (six categories), and TNM stage (I–IV). Social status was classified according to registry categories (employed, retired, unemployed). Diagnostic circumstances were categorized as screening-detected, symptomatic presentation, or other diagnostic pathways recorded in the registry. Predictors were selected based on clinical relevance and registry availability. Categorical variables were encoded using one-hot encoding, and age was standardized using z-score normalization. The same preprocessing pipeline was applied across all analyses.

### 2.3. Machine Learning Phenotyping and Survival Analysis

Unsupervised phenotyping was performed using k-means clustering with k-means++ initialization and Euclidean distance. Because most predictors were categorical, clustering was applied to a one-hot encoded representation of the data. We acknowledge that k-means is primarily designed for continuous variables and that the resulting clusters should therefore be interpreted as pragmatic data-driven groupings rather than definitive latent classes. Solutions with two to six clusters were evaluated, and the optimal number was selected using the silhouette coefficient. The two-cluster solution produced the highest silhouette score (0.137) and was retained for subsequent analyses. Clusters were interpreted as pragmatic data-driven phenotypes. Although the silhouette score indicated modest separation, the two-cluster solution was retained because it showed the best relative performance among the tested solutions and yielded clinically interpretable survival differences. Given the low silhouette score, clustering results were interpreted as exploratory and primarily descriptive rather than representing stable or clinically definitive phenotypes.

Phenotypes were summarized using descriptive statistics. Age differences were tested using independent t-tests and categorical variables using chi-square tests. Survival differences were assessed using Kaplan–Meier curves and the log-rank test. A Cox proportional hazards model with cluster membership as the predictor (Cluster 0 as reference) estimated hazard ratios (HRs) and 95% confidence intervals (CIs). Model discrimination was summarized using the concordance index (C-index), and the proportional hazards assumption was assessed using Schoenfeld residuals. No major violation of the proportional hazards assumption was detected.

### 2.4. Random Survival Forest Risk Prediction and Clinical Utility

Individualized short-term mortality risk was estimated using a random survival forest (RSF) model with the same predictors and preprocessing steps. The model used 500 trees with min_samples_leaf = 15, min_samples_split = 10, and max_features = “sqrt”, with parallel computation and a fixed random seed. To ensure robust model evaluation and minimize overfitting, a multi-step validation framework was applied. The dataset was temporally split into a training cohort (2019–2021) and an independent test cohort (2022). Within the training cohort, model stability was assessed using fivefold cross-validation. In addition, bootstrap resampling (1000 iterations) was used to estimate optimism and obtain optimism-corrected performance estimates. For each patient, the model generated a survival function Ŝ(t). Predicted 1-year mortality risk was defined as:

risk_1_y = 1 − Ŝ (12 months).

Model performance was evaluated using multiple complementary metrics. Discrimination was assessed using the concordance index (C-index). Prediction accuracy was evaluated using the Brier score at 12 months. Model calibration was assessed by comparing the predicted and observed risks across quantile-based groups, with observed probabilities estimated using Kaplan–Meier methods to account for censoring. For benchmarking purposes, two comparator models were developed: a penalized Cox proportional hazards model using the same predictors and a TNM stage–only model. Differences in model discrimination were evaluated using bootstrap resampling to estimate confidence intervals for the difference in C-index between models. Predicted risks were categorized into low, intermediate, and high groups using the 25th and 75th percentiles of the risk distribution. These thresholds were selected as a data-driven approach to define relative risk groups and should be interpreted primarily as exploratory rather than clinically predefined cut-offs. Prognostic separation across groups was evaluated using Kaplan–Meier analysis and Cox regression with the low-risk group as reference. Clinical utility was assessed using decision-curve analysis across threshold probabilities from 0.05 to 0.60 and compared with treat-all, treat-none, and a TNM stage-based rule (stage ≥ III).

### 2.5. Software

Analyses were conducted in Python (v3.12) using scikit-survival, scikit-learn, lifelines, pandas, NumPy, matplotlib, and seaborn within the Google Colaboratory environment.

## 3. Results

### 3.1. Machine Learning Phenotyping of Lung Cancer Patients

We applied k-means clustering to eight baseline predictors (age, sex, nationality, residence, social status, diagnostic circumstances, histology, and TNM stage). After one-hot encoding of categorical variables and z-score standardization of age, silhouette analysis across k = 2–6 indicated that k = 2 provided the best separation (silhouette = 0.137), yielding two ML-derived phenotypes. However, the silhouette score was low (0.137), indicating weak cluster separation; therefore, the identified phenotypes should be interpreted as exploratory and primarily descriptive.

#### 3.1.1. ML-Derived Clusters and Baseline Profiles

The final solution classified patients into Cluster 0: *n* = 7692 (56.2%) and Cluster 1: *n* = 5993 (43.8%). Cluster 0 represented an older phenotype (70.3 ± 5.8 years) that was more urban and had higher representation of Social Status Group 2, with slightly more Stage III disease. Cluster 1 was younger (56.1 ± 7.3 years), more rural and socially disadvantaged, with a marginally higher proportion of Stage IV disease. Sex distribution was similar across clusters. Full baseline characteristics are shown in Table 1.

#### 3.1.2. Survival by Phenotype

**Figure 2:**
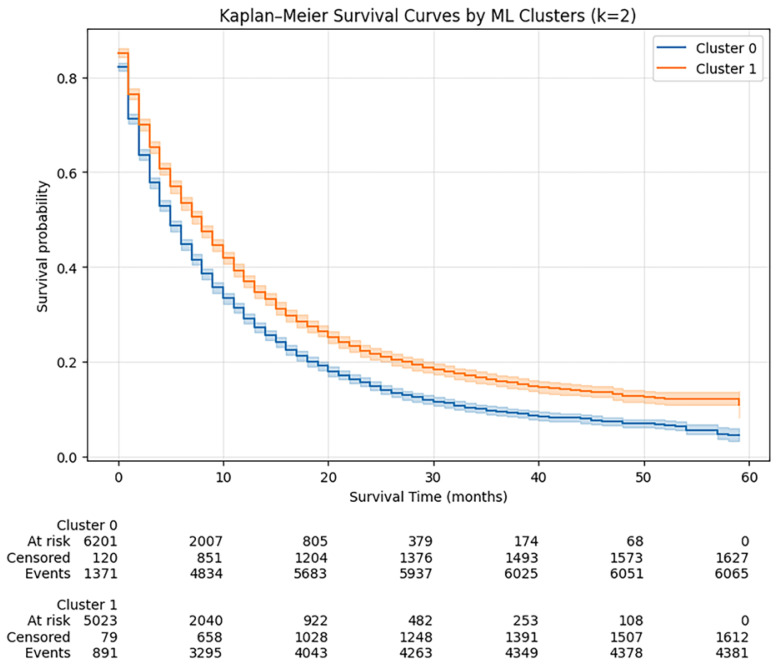
Kaplan–Meier overall survival curves for ML-derived phenotypes (Cluster 0 and Cluster 1) with 95% confidence intervals and numbers at risk. Differences between clusters were significant (log-rank *p* < 0.001).

Kaplan–Meier analysis demonstrated significantly different survival between phenotypes (log-rank χ^2^ = 141.29, *p* < 0.001). Median OS was 5 months in Cluster 0 versus 8 months in Cluster 1. One-year OS was 29.1% (Cluster 0) vs. 36.9% (Cluster 1); three-year OS 9.5% vs. 15.9%; five-year OS 4.4% vs. 10.9% (see Figure 2 and Table 2). In a univariable Cox model, Cluster 1 had lower mortality compared with Cluster 0 (HR = 0.79, 95% CI 0.76–0.82; *p* < 0.001), with limited discrimination when using phenotype alone (C-index = 0.53). This low discrimination indicates that clustering alone has limited prognostic value.

### 3.2. ML-Based 1-Year Mortality Prediction and Risk Stratification

A random survival forest (RSF) model was developed in the training cohort and evaluated in an independent temporal test cohort, generating individualized 1-year mortality risk estimates defined as risk_1_y = 1 – Ŝ (12 months). Predicted risks varied widely across patients, from very low (<0.05) to very high (>0.90), indicating substantial prognostic heterogeneity.

#### 3.2.1. Three-Level Risk Groups

In the full cohort, percentile-based cutoffs (25th and 75th percentiles) were used to define low, intermediate, and high risk groups, corresponding approximately to 25%, 50%, and 25% of patients. In the temporal test cohort, applying cutoffs derived from the training cohort resulted in 880 patients classified as low risk, 1422 as intermediate risk, and 597 as high risk.

#### 3.2.2. Survival Separation and Effect Sizes

**Figure 3:**
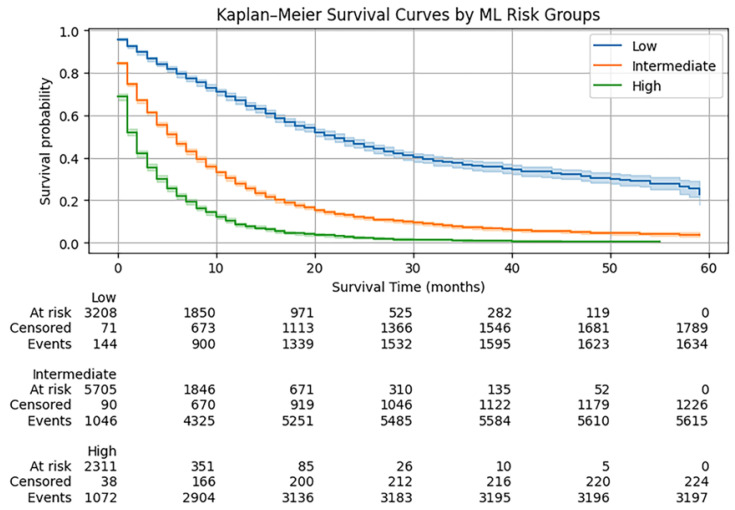
Kaplan–Meier overall survival curves stratified by ML-derived 1-year mortality risk groups (low, intermediate, and high), with 95% confidence intervals and numbers at risk. Clear separation between groups was observed (log-rank *p* < 0.001).

Kaplan–Meier curves showed strong and graded separation across ML risk groups (Figure 3), confirmed by a highly significant log-rank test (χ^2^ = 3427.69, *p* < 0.001). In Cox regression (low as reference), the intermediate group had HR = 2.86 (95% CI 2.70–3.02; *p* < 0.001), and the high group HR = 5.66 (95% CI 5.32–6.02; *p* < 0.001) (Table 3). Discrimination of the ML risk grouping was C-index = 0.67.

#### 3.2.3. Model Validation and Performance

The RSF model demonstrated stable performance across validation strategies. In the temporal test cohort, the C-index was 0.679, similar to the training cohort (0.679), indicating minimal overfitting.

Fivefold cross-validation within the training cohort yielded a mean C-index of 0.660 (SD 0.005). Bootstrap resampling showed an optimism of 0.017, resulting in an optimism-corrected C-index of 0.663. These findings suggest good internal validity and stability of the model.

### 3.3. Model Comparison

In the temporal test cohort, the RSF model outperformed both comparator models. The penalized Cox model showed lower discrimination (C-index = 0.544), while the TNM stage-only model achieved a C-index of 0.606.

The difference in discrimination between RSF and Cox was 0.136 (95% CI 0.120–0.150), and was 0.073 (95% CI 0.061–0.085) between RSF and TNM, supporting the incremental value of the machine learning approach.

### 3.4. Prediction Accuracy and Calibration

At 12 months, the RSF model demonstrated improved prediction accuracy compared with both comparator models, with a Brier score of 0.169 versus 0.212 for both Cox and TNM models.

**Figure 4:**
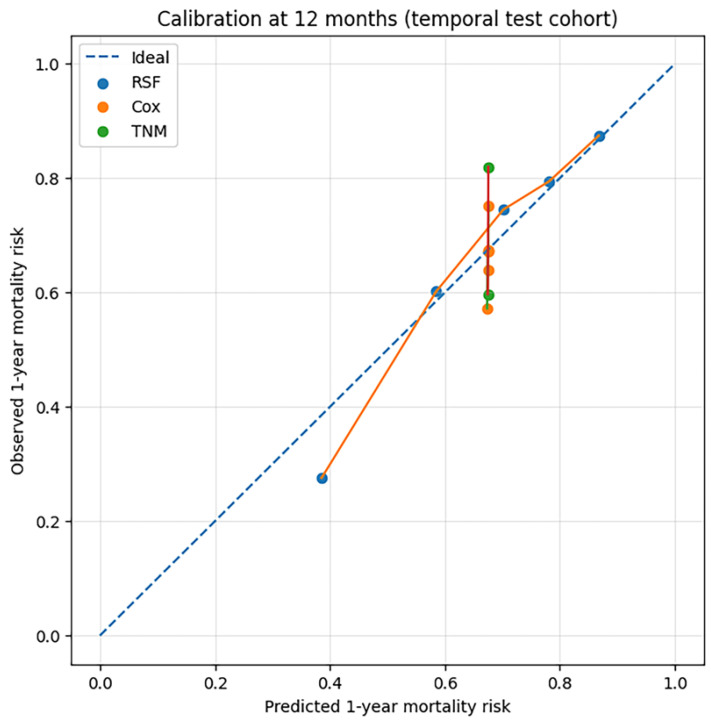
Calibration plot for 1-year mortality prediction in the temporal test cohort. The RSF model showed good agreement between the predicted and observed risks, with slight overestimation in the lowest-risk group.

Calibration analysis showed good agreement between the predicted and observed risks across risk groups, although slight overestimation was observed, particularly in the lowest predicted risk range (Figure 4).

### 3.5. Clinical Utility Compared with TNM Staging

When evaluated in the full cohort, TNM stage alone showed lower discrimination (C-index = 0.64) compared with the ML-derived risk groups (C-index = 0.67). However, these estimates reflect apparent performance and should be interpreted cautiously.

In the temporal test cohort, the RSF model demonstrated higher discrimination (C-index = 0.679) than both the TNM stage-only model (C-index = 0.606) and the penalized Cox model using the same predictors (C-index = 0.544).

**Figure 5:**
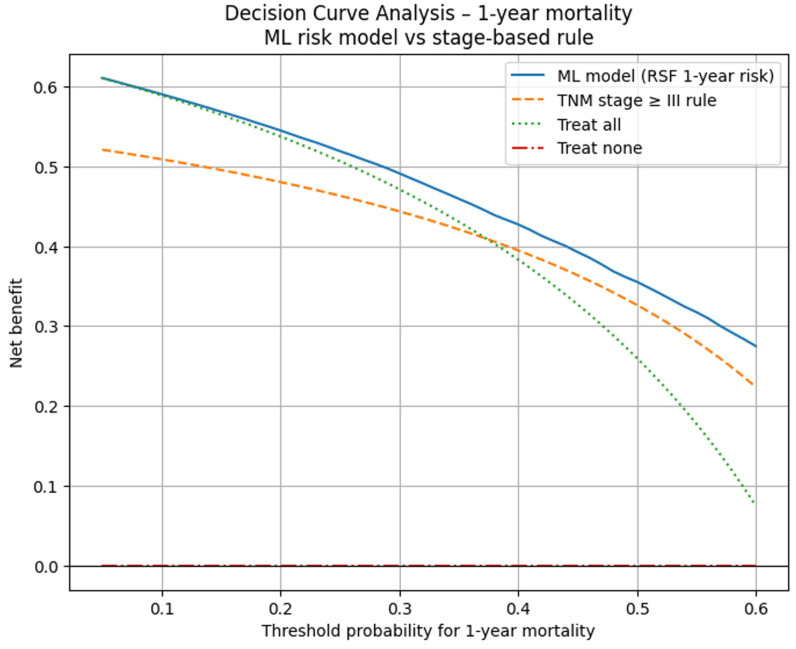
Decision-curve analysis comparing the ML-based 1-year mortality risk model with TNM stage-based classification.

Decision-curve analysis indicated a higher net benefit for the ML model than the stage-based rule (stage ≥ III) and “treat-all/treat-none” strategies across ~10–50% 1-year risk thresholds (Figure 5). These thresholds correspond to clinically plausible decision ranges for identifying patients who may benefit from intensified monitoring or supportive care. The model demonstrated higher net benefit across this range, suggesting potential clinical relevance, although further external validation is required before routine implementation.

## 4. Discussion

This study used a large national lung cancer registry from Kazakhstan to develop and internally validate a pragmatic machine learning framework for 1-year mortality risk stratification. The framework combined exploratory unsupervised phenotyping, RSF-based survival prediction, temporal validation, calibration assessment, and decision-curve analysis. The main finding is that the RSF model showed stable internal performance and improved discrimination and prediction accuracy compared with both TNM stage alone and a penalized Cox model using the same predictors in this dataset. First, the unsupervised clustering analysis identified two patient groupings with different demographic profiles and survival outcomes. However, these findings should be interpreted cautiously. The silhouette score was low (0.137), indicating weak cluster separation, and phenotype-only discrimination was limited (C-index = 0.53). This contrasts with a previously published study in which unsupervised clustering methods, such as two-step and hierarchical clustering, demonstrated moderate cluster separation and significant survival differences [19]. In our study, the lower silhouette score may indicate greater overlap between groups, which could partly reflect the heterogeneity of a national registry cohort and the limited set of routinely available variables.

Second, the RSF model produced individualized 1-year mortality risks that were translated into three pragmatic risk groups. The clear stepwise separation in survival across these groups indicates that the model captured clinically relevant short-term prognostic differences. In temporal validation, the C-index was 0.679 in both the training and test cohorts. Fivefold cross-validation showed similar performance (mean C-index = 0.660, SD = 0.005), and bootstrap optimism correction produced an optimism-corrected C-index of 0.663. Together, these findings suggest limited overfitting and support the internal robustness of the model. These findings are consistent with prior studies suggesting that tree-based survival models can capture nonlinear relationships and complex interactions in clinical data [20]. Several contemporary oncology studies have reported that random survival forest models may provide improved predictive performance compared with traditional Cox regression models, although the results vary depending on dataset characteristics and feature structure [21,22].

In addition, the RSF model demonstrated improved prediction accuracy, with a 12-month Brier score of 0.169 compared with 0.212 for both the penalized Cox and TNM-only models. Calibration analysis showed generally good agreement between the predicted and observed 1-year mortality risks, although risk was slightly overestimated in the lowest-risk group. Such miscalibration patterns have been reported in prediction model validation studies and may reflect differences in case-mix or the relative scarcity of events in lower-risk strata [23]. Accurate calibration is critical for clinical decision-making, as miscalibration may lead to inappropriate risk stratification and suboptimal treatment allocation [24].

Notably, the magnitude of improvement in discrimination observed in this study appears larger than typically reported. Prior studies have generally found only modest differences between machine learning and Cox-based models, with gains often being small and context-dependent [17,25,26,27]. The larger difference observed here may reflect specific characteristics of the registry dataset, including the limited number of predictors and the presence of nonlinear relationships better captured by tree-based methods. In addition, the penalized Cox model produced relatively homogeneous risk estimates across patients, suggesting limited ability to capture nonlinear relationships and interactions in this dataset. Decision-curve analysis suggested that the RSF model may provide greater net benefit than TNM-based classification across clinically plausible 1-year mortality thresholds. In this context, higher predicted risk could support intensified follow-up, earlier supportive or palliative care referral, or prioritization for multidisciplinary review. Decision-curve analysis is now widely recommended as a standard method for evaluating the clinical utility of prediction models, because it quantifies the balance of benefits (true positives) and harms (false positives) across different risk thresholds that correspond to real-world decision points [28]. However, these thresholds should be interpreted as decision-analytic assumptions rather than established clinical cutoffs, and their acceptability should be evaluated prospectively with clinicians and health-system stakeholders. These findings align with the broader shift in modern clinical practice toward risk-adapted and individualized management strategies, where quantitative risk estimation is used to guide clinical decision-making and balance the benefits of early detection with the risks of overtreatment [29].

Finally, this work adds region-specific evidence from a Central Asian cohort, an area that is under-represented in lung cancer machine learning research. Most published ML survival models in lung cancer have been developed in East Asian, European, or North American populations, often using institutional cohorts or clinical trial datasets that may not reflect the sociodemographic and health-system context of Kazakhstan and neighboring countries [30]. Our national registry-based approach therefore broadens the geographic scope of ML prognostic research in lung cancer.

### 4.1. Strengths and Limitations

A major strength of this study is the use of a large, real-world, national registry with 13,685 patients, which increases the precision of estimates and enhances generalizability within Kazakhstan. Real-world data are essential for building clinically relevant prediction models as they capture the heterogeneity in patient characteristics, treatment access, and care pathways that is often absent from clinical trials. Another strength is the integrated design: we combined unsupervised clustering, survival modeling, risk grouping, and decision-analytic evaluation in a single analytic framework, which aligns with several current best practice recommendations, although some elements such as external validation and formal cluster stability assessment were not available in this study [31]. The model should therefore be interpreted as a pragmatic registry-based baseline risk stratification model rather than a comprehensive individualized prognostic tool. The 25th and 75th percentile cutoffs used to define low, intermediate, and high risk groups were data-driven and sample-dependent. They were selected to facilitate the interpretation and presentation of results, but should not be considered clinically validated thresholds. Future studies should explore clinically meaningful or externally validated cut points.

The study also has limitations. First, the analysis relied on registry variables only and lacked detailed information on performance status, comorbidities, smoking history, treatment details, and molecular biomarkers, all of which may influence survival and predictive performance. Contemporary survival models in lung cancer increasingly integrate clinical, radiologic, and molecular features to capture different dimensions of risk [32]. In addition, registry-based data may be subject to variability in data completeness and accuracy across institutions, and residual confounding cannot be excluded. Second, although we performed temporal validation, fivefold cross-validation, and bootstrap optimism correction, external validation in an independent dataset was not available. Therefore, the model’s transportability beyond this registry and health-system context remains uncertain and should be assessed in future external cohorts.

Formal cluster stability analysis was not performed; therefore, the stability of these exploratory phenotypes across resampling or external cohorts remains uncertain. Although random survival forests were selected because of their strong performance in censored survival data and their ability to capture nonlinear interactions, other machine learning approaches were not systematically compared in this study, and different algorithms may perform better depending on the dataset and feature structure. Comparative studies have shown that no single algorithm is universally superior and that different methods may perform better in different data contexts [33]. Fourth, the registry-level staging information was based on the TNM system in use during the study period, and we did not re-stage patients according to the recently introduced 9th edition, which aims to refine prognostic discrimination, especially in advanced disease [34].

Finally, although decision-curve analysis provides a powerful framework for evaluating clinical utility, it relies on assumptions about the range of threshold probabilities that reflect real-world decision-making. The choice of 10–50% as the primary window was informed by general practice and the DCA literature, but future work should validate the acceptability of these thresholds with local clinicians and health policy stakeholders [35].

### 4.2. Implications for Clinical Practice

Our findings have several implications for the clinical management of lung cancer in Kazakhstan and similar settings. First, the exploratory clustering analysis illustrates that routinely collected registry variables can describe broad patient groupings with different survival patterns. However, because cluster separation and phenotype-only discrimination were limited, these groupings should be interpreted cautiously and should not be used as standalone clinical decision tools. Comparable studies in NSCLC and other cancers have suggested that such phenotypes may help identify high-risk groups who could benefit from closer surveillance or more aggressive treatment strategies [19].

Second, the three-level RSF-based risk score offers a pragmatic approach that may eventually be evaluated for integration into electronic medical records after external validation and prospective implementation testing. Such a tool could support risk stratification at the time of diagnosis, helping to identify patients at higher short-term mortality risk. In this context, higher predicted risk may justify intensified follow-up, earlier referral to supportive or palliative care, or prioritization for multidisciplinary review, whereas lower-risk patients may avoid unnecessary overuse of limited resources. Similar ML risk tools have been proposed to support individualized treatment decisions, such as selecting candidates for intensive therapy, prioritizing early palliative care, or tailoring follow-up intensity based on predicted survival [36].

Third, the DCA results suggest that ML-guided risk stratification could improve net clinical benefit over TNM staging alone by better aligning intensive interventions with actual risk. Decision-curve analysis is increasingly recommended alongside traditional performance metrics (C-index, AUC, calibration) when evaluating prediction models, because it directly addresses whether a model is likely to do more good than harm in practice [28]. In resource-constrained health systems, such as those in parts of Central Asia, tools that improve the targeting of limited diagnostic and treatment capacity may have particularly high value.

At the same time, successful implementation will require attention to workflow integration, clinician training, and interpretability. Frameworks for deploying ML prediction models in clinical settings emphasize the need for clear user interfaces, governance structures, and the evaluation of impact on patient outcomes and equity, rather than relying solely on statistical performance in retrospective data [31]. Our risk groups were intentionally simple (three categories, based on routinely available predictors) to facilitate adoption and communication; however, future implementation studies will be needed to confirm usability and acceptability among clinicians and patients.

### 4.3. Future Research

Future work should focus on external validation of our ML models and risk groups in independent cohorts within Kazakhstan and in other countries in Central Asia and Eastern Europe. Cross-regional validation is critical to determine whether ML predictors learned in one health system generalize to populations with different demographic and treatment patterns [36].

Second, integrating additional data sources—such as imaging-derived radiomics, laboratory biomarkers, performance status, and genomic information—may further improve the discrimination and calibration of survival predictions, as shown in recent multi-domain and multi-omic prognostic models in lung cancer [37]. Third, more advanced ML approaches, including semi-supervised learning, deep survival models, and foundation-model-based pipelines, could be explored to leverage both labeled and unlabeled data and to strengthen robustness across institutions [38]. Fourth, prospective impact studies are needed to evaluate whether using ML-derived risk groups to guide clinical decisions actually improves patient-centered outcomes, such as quality of life, timely access to palliative care, or reduction in avoidable hospitalizations. Methodological work on decision-analytic evaluation of prediction-guided care pathways suggests that combining DCA with pragmatic trials or quasi-experimental designs may offer a rigorous way to quantify real-world benefits and harms [35].

Future implementation research should also explore how the three-level ML risk groups could be integrated into electronic health record systems to automatically generate risk stratification at the time of diagnosis. Such integration could enable real-time decision support for clinicians and facilitate prospective studies evaluating whether ML-guided risk stratification improves clinical outcomes, resource allocation, and early referral to supportive or palliative care services. Finally, given ongoing updates in TNM staging (including the introduction of the 9th edition), future research could examine how ML-derived risk groups interact with refined stage categories and whether combined TNM–ML models provide a further step forward in prognostication.

## 5. Conclusions

In this national lung cancer cohort from Kazakhstan, an RSF-based model using routinely collected registry variables demonstrated stable internal performance for 1-year mortality risk stratification. The model showed consistent discrimination in temporal validation, cross-validation, and bootstrap optimism correction, and outperformed both TNM stage alone and a penalized Cox model in the temporal test cohort.

Calibration and Brier score analyses further supported the reliability of the RSF model for short-term risk prediction within this dataset. The unsupervised clustering component identified descriptive patient groupings, but these should be interpreted as exploratory given the low silhouette score and limited phenotype-only discrimination.

Overall, these findings suggest that pragmatic ML-based risk stratification may provide additional prognostic information beyond conventional staging. However, external validation and prospective implementation studies are required before clinical use.

## Figures and Tables

**Table 1:** Sociodemographic and clinical characteristics of ML-derived lung cancer phenotypes.

Variable	Cluster 0 (*n* = 7692)	Cluster 1 (*n* = 5993)
Age, mean ± SD	70.3 ± 5.8	56.1 ± 7.3
Sex (% male)	78.4%	81.0%
Nationality (%)		
- Kazakh	47.9%	57.1%
- Russian	33.9%	27.4%
- Other	18.2%	15.4%
Residence (%)		
- Urban	66.2%	57.4%
- Rural	33.8%	42.6%
Social Status (%)		
- Employed	19.6%	47.9%
- Retired	75.5%	2.7%
- Unemployed	4.8%	49.4%
Diagnostic Circumstances (%)		
- Screening	34.1%	32.8%
- Symptomatic	56.2%	56.8%
- Other	9.7%	10.4%
Histology (%)		
- Adenocarcinoma	24.6%	31.1%
- Squamous cell carcinoma	33.2%	32.3%
- Large cell carcinoma	2.2%	3.0%
- Small cell carcinoma	8.4%	10.3%
- NSCLC, not otherwise specified	29.7%	20.5%
- Other lung cancer	1.9%	2.8%
Stage (%)		
- Stage I	6.5%	7.8%
- Stage II	18.9%	19.7%
- Stage III	49.3%	44.2%
- Stage IV	25.3%	28.3%

**Table 2:** Survival outcomes by ML-derived phenotype.

Survival Metric	Cluster 0 (*n* = 7692)	Cluster 1 (*n* = 5993)
Median OS (months)	5.0	8.0
1-year OS (%)	29.1%	36.9%
3-year OS (%)	9.5%	15.9%
5-year OS (%)	4.4%	10.9%
Log-rank χ^2^	141.29	—
*p*-value	<0.001	—

**Table 3:** Hazard ratios for mortality across ML-derived risk groups.

Risk Group	Hazard Ratio (HR)	95% CI	*p*-Value
Low (reference)	1.00	–	–
Intermediate	2.86	2.70–3.02	<0.001
High	5.66	5.32–6.02	<0.001

## Data Availability

The data presented in this study are available on request from the corresponding author. The data are not publicly available due to privacy and ethical restrictions.
